# Meetings of Medical Societies in Richmond

**Published:** 1875-09

**Authors:** 


*Meetings of Medical Societies in Richmond.*

The sixth session of the Medical Society of Virginia will convene in Richmond at 7½ P. M., Wednesday, October 20th, 1875, in a hall to be designated in a circular to be issued about October, 1st. Every indication is that this will be a "grand rally" of the profession of the State. Every regular physician in Virginia who has not yet connected himself with the Society—organized to promote scientific and general professional interests—is earnestly requested at once to forward his application for membership, endorsed by any member of the Society. Committees are urged to have their reports ready for presentation; and all members having papers or reports of cases in course of preparation, or in contemplation, are requested to have them ready for handing directly to the Committee on Publication immediately after presentation to the Society.

The Association of Medical Officers of the Confederate Army and Navy will convene at the same place at 11 A. M., Tuesday, October 19th, 1875. All communications for this Association should be addressed to the President, Dr. S. P. Moore, Richmond, Virginia, or to the Secretary, Dr. S. H. Stout, Atlanta, Georgia.

Every effort will be made to secure the usual reduced fare over railroads, boat lines, etc., which, as far as practicable, will be announced in the forthcoming circular. It is a pleasure, however, to announce at this early day that the proprietor of St. James Hotel—one of the very best in the State in every respect—has agreed to charge members and delegates with their families, who may attend, only two dollars per diem during the period in which either or both of these Societies may be in session.

We clip the above from the *Virginia Medical Monthly.* To all who can spare the time to attend these meetings, we feel authorized in saying that the profession of Richmond will spare no pains or expense to make their visit pleasant and profitable. P.

